# An Uncommon Occurrence after Amputation

**Published:** 1800-01

**Authors:** G. Rowlands

**Affiliations:** Chester


### *To the Editors of the Medical and Phyfical Journal.*

GENTLEMEN,

IF the following cafes merit a place in the Medical and Phyfical Journal, you will oblige me by inferting them in your next Number. I have the honour to be, GENTLEMEN,

Your moft obedient humble fervant,

*Chefter, Oct.* 19, 1799. G. ROWLANDS.

---

### *An uncommon Occurrence after Amputation.*

ON September the 9th, 1794, Robert Jones, a collier, was drawn up with great velocity out of a pit, forty yards deep, by the fore arm, which was nearly feparated from the elbow, by being drawn between the rope and the wheel. The accident happened early in the morning; and a furgeon in the neighbourhood applied a tourniquet about four inches below the fhoulder. He was brought to the Infirmary at fix in the evening, and his arm was immediately amputated above the ligature, which had been tight on all day. The man bore a journey of ten miles in a cart, with his arm in that fhattered condition, and fuftained the operation without either fainting or complaining; he took an opiate, and had a better night than could have been expected.

The next day a clyfter was adminiftered, and he took faline draughts, with fifteen drops of vin. ant. in each, to remove a flight degree of fever: he continued the opiate every night. On the 13th, I looked at the ftump, and had the fatisfaction to find it perfectly united in every part, but where the fingle ligature hung out, only one artery having appeared. On the 22d the ligature feparated; and on the 25th, all dreffings, except a little lint and the roller, were left off.

The man had walked about the ward for many days, his appetite being good, and his nights undifturbed.—On the 27th, about three in the morning, I was defired to come to him immediately, the meffenger at the fame time informing me that he was bleeding to death.—I haftened to him, and found the poor man in a miferable condition. The tourniquet had been applied by Mr. Manning, the houfe apothecary; but as the hæmorrhage came on in the middle of the night, he had loft fo much blood before a difcovery was made, that the bed was wet through, and the blood flowed acrofs the ward After removing the bloody things from about the ftump, I difcovered the caufe of this extraordinary accident, to be an extenfive mortification of the artery and integuments covering it. The union of of the ſtump continued complete; the ſkin having ſeparated in a ſlough, about an inch from the edge of the cicatrix. I made a ligature on the artery, and doſils of lint were applied to the mouth of it. The wound was dreſſed with mild digeſtive; every means had been uſed, and were ſtill continued, to recover him from the low, fainting ſtate to which he was reduced; and a perſon was directed to watch him conſtantly, and to make a ſlight compreſſion on the end of the veſſel. 28th, No appearance of blood. 29th, In the night, a ſecond ſloughing of the artery took place, and the ligature came off. The tourniquet was tightened as expeditiouſly as poſſible; but ſo great was the loſs of blood, that, in his reduced ſtate, it had nearly deſtroyed him. As ſoon as he was a little recovered, I made an inciſion in the direction of the artery to the axilla, and put a ligature on the veſſel, as high up as poſſible. The wound was dreſſed as before, and moderate preſſure was continued by a careful aſſiſtant. Nothing further occurred worthy of obſervation; the wound healed ſlowly, but eventually, ſo as to leave a very good ſtump; and he was diſcharged from the Infirmary in good health on the 28th of November following.

